# Evidence-Based Efficacy of Autologous Grated Cartilage in Primary and Secondary Rhinoplasty

**Published:** 2017-05

**Authors:** Ali Manafi, Ali Kaviani, Zahra Sadat Hamedi, Afsaneh Rajabiani, Navid Manafi

**Affiliations:** 1 Department of Plastic Surgery, Iran University of Medical Sciences, Tehran, Iran;; 2 Department of Plastic Surgery, Shiraz University of Medical Sciences, Tehran, Iran;; 3 Department of Plastic Surgery, Shahid Beheshti University of Medical Sciences, Tehran, Iran;; 4 Department of Pathology, Tehran University of Medical Sciences, Tehran, Iran

**Keywords:** Autologous, Cartilage, Graft, Rhinoplasty

## Abstract

**BACKGROUND:**

There are numerous methods to mold and shape cartilage grafts for use in rhinoplasty. Each technique has advantages and disadvantages. We are going to introduce a new method for cartilage shaping with long lasting effects confirmed by follow up examination and pathologic evaluation.

**METHODS:**

Grated cartilage was used in 483 patients. For 89 cases, it was wrapped in fascia and in 394 patients, used as a filler per se or in contiguity with solid structural grafts. In 51 patients, the operation was primary rhinoplasty and 432 cases, underwent secondary rhinoplasty. Postoperatively, there was a mean follow up of 2.8 years. Graft viability, and capability to maintain almost original volume, and general durability were assessed.

**RESULTS:**

Out of 483 patients, only 23 cases (4.7%) needed later correction. In 11 cases (2%), it was due to overcorrection and some minor imperfections. In the rest 12 cases (2%), there was a need for more augmentation probably due to some degree of graft resorption. Three cases of these 12 patients, were corrected by outpatient shaved cartilage injection.

**CONCLUSION:**

According to the very low revision rate (less than 5%), we strongly recommend our grated cartilage graft for use in primary and secondary rhinoplasty. Our study showed that patient and surgeon`s satisfaction can be achieved with a high degree of confidence.

## INTRODUCTION

Using cartilage grafts in primary and repetitive rhinoplasty is mandatory; because maintenance or reinforcement of the major supporting structures is fundamental for aesthetic and functional purposes.^1^ Basically, there are two main categories of cartilage grafts used in rhinoplasty including structural and filler types. Structural type grafts are solid, strong blocks of hyaline cartilage to fortify different parts of the nose. Filler type cartilage grafts are pieces of molded hyaline or elastic cartilage to fill small imperfections for camouflage. They have no internal memory or structural strength. Various methods of cartilage molding have been stated in the literature. One of the first recommended manners to mold the cartilage was crushing introduced in 1985-1986.^2^

During crushing, multiple uncontrolled fractures are made to overcome internal memory of the cartilage and reshape it. Then, crushed cartilage is inserted beneath the nasal envelope to fill small defects of soft tissue. After crushing, the proportion of surviving chondrocytes is 10-30% depending on the severity of the external forces.^3^ So the majority of the graft bulk will be resorbed and the filling material is lost. In comparison, cutting the cartilage graft, imposes much less trauma and necrosis to the chondrocytes and better survival of the graft.^3^ That’s why, crushed cartilage was replaced by diced cartilage in 2000 first described by Erol as the Turkish delight theory.^4^ Afterwards, Daniel and Calvert, popularized the diced cartilage graft usage in rhinoplasty in 2004.^5^ Daniel and Calvert proved that diced cartilage can be used separately or wrapped in fascia with an ignorable risk of graft resorption.^5^

Nowadays, dicing is the preferred method of cartilage molding to use as an autologous filling material. Dicing process involves manual cutting of the cartilage into pieces less than 1 mm.^6^ It is time consuming and cartilage pieces are not homogenous. On the other hand, dicing is operator dependent and for thin skinned noses, there is a risk of palpability or visibility if not wrapped in fascia. We recommend our cartilage grating device (innovated). It is fast and easy to use and we have confirmed evidence of chondrocyte viability with very little revision rate.^7^ This study determined the evidence-based efficacy of autologous grated cartilage graft in primary and secondary rhinoplasty.

## MATERIALS AND METHODS

Applying grater device for molding and cutting rib or conchal cartilage to use it as a filler graft, has been our routine procedure in secondary rhinoplasty, since7 years ago. In another published paper,^7^ we have clearly defined the durability and viability of grated cartilage grafts in rabbit. Our tendency to grate cartilage grafts was invigorated when the senior author realized that crushed cartilage has a high resorption rate and abandoned using it. Since about a decade later, when diced cartilage was introduced, it came into mind that using a device to dice, cartilage grafts, converts dicing process to a faster and easier procedure.^7^ The principal question here is “what is the effect of grating on chondrocyte viability?” To answer this question, we designed this study. We planned to answer, according to the histologic evidence and not solely by clinical data. In this study, we used grated cartilage as the filler graft of choice in 483 secondary and primary cases during 7 years. Our patients were between 19-65 years old (mean=34 y/o) (Table 1). 

**Table 1:** Summary of graft usage and results

**Gender**	**Female (425)**	**Male (58)**
Graft usage form	Bare (394)	Wrapped in fascia (89)
Results	Satisfactory (460, 95.3%)	Further intervention (23, 4.7%)

**Fig. 1:**
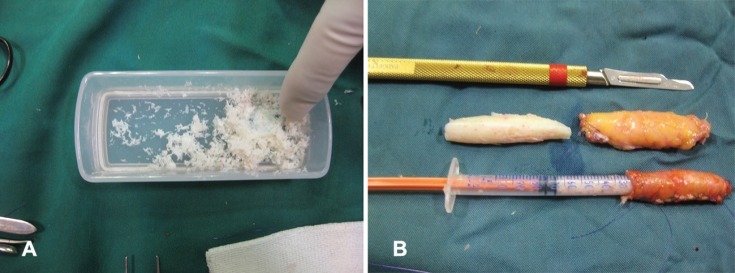
**A:** Grated cartilage products. **B: **Grated cartilage wrapped in fascia

**Fig. 2:**
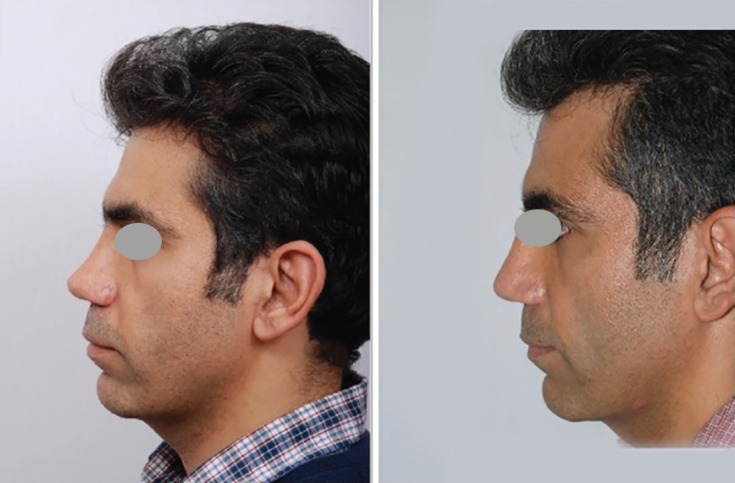
Forty years old man complaining from minor over resection of nasal dorsum during his rhinoplasty operation. After vigorous skin massage,^12^ revision rhinoplasty with liberal augmentation of nasal dorsum with autologous grated cartilage. It shows some degrees of overcorrection at the nasal dorsum and radix

In 89 (18%) patients, it was wrapped in fascia (Figure 1). Rectus abdominis fascia was applied in 73 (15%), deep temporal fascia in 5 (1%) and rib perichondrium in 11 (2%) cases. For 31 cases (6%), grated cartilage was used solely and in the rest 363 cases (75%), it was applied in contiguity with other solid structural rib grafts to fill small defects. At the beginning of the study, we were not sure about graft survival and several cases were overcorrected (Figure 2). Later, many of them needed surgical reduction and debulking. 

**Fig. 3:**
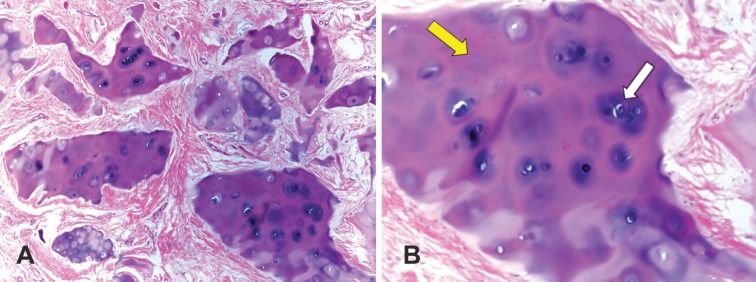
The histologic figure of resected cartilaginous tissue from the overcorrected parts of nasal dorsum and radix in the case of Figure 2, during revision surgery. The cartilaginous tissue is vital as evidenced by the presence of active nuclear mitotic figures. This grafted cartilage shows evidence of growth and proliferation (**A:** ×40 and **B**: ×100), composed of homogenous matrix (yellow arrow) and frequent chondrocyte population (white arrow). The chondrocytes are round, but showing straight outlines in contacts with each other. The cartilage tissue fragments are surrounded by vascular fibrous stroma

**Fig. 4:**
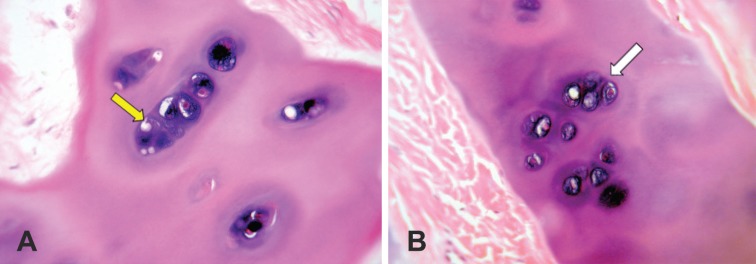
The chondrocytes are located in cavities in the matrix, called cartilage lacunae. Each lacunae is generally occupied by a single Cell. In active proliferative process, the lacunae may contain two, four, or eight cells (yellow and white arrows) (**A** and **B**: ×100

**Fig. 5:**
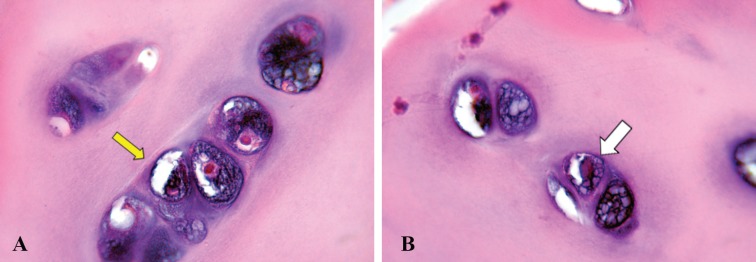
The cartilage lacunae are actually artificial gaps, formed by shrinkageof the cell cytoplasm, during the staining and setting of the tissue (yellow arrow). The chondrocytes have one or two prominent nuclei with usual intranuclear network (white arrow) (**A** and **B:** ×400

During revision, some parts of resected cartilage were submitted for pathologic evaluation. Chondrocyte regenerative capability leading to graft survival was assessed (Figures 3-5). Out of all 483 cases, 23 cases (4.7%) needed further intervention. Totally, 460 patients (95.3%) had satisfactory follow up period and never needed any corrective procedure. In the revision surgery group, 11 cases were due to overcorrection and prominence in the radix or dorsum. Five cases had asymmetry or deviation at radix or dorsum or tip area. Four cases with tip deformity and 3 ones with a concavity or dimpling in the dorsum or sidewalls completed this group of patients. The first 20 cases underwent revision surgery and the last 3 patients with small imperfections were corrected by shaved cartilage injection in an outpatient session.^8^

## RESULTS

**Fig. 6:**
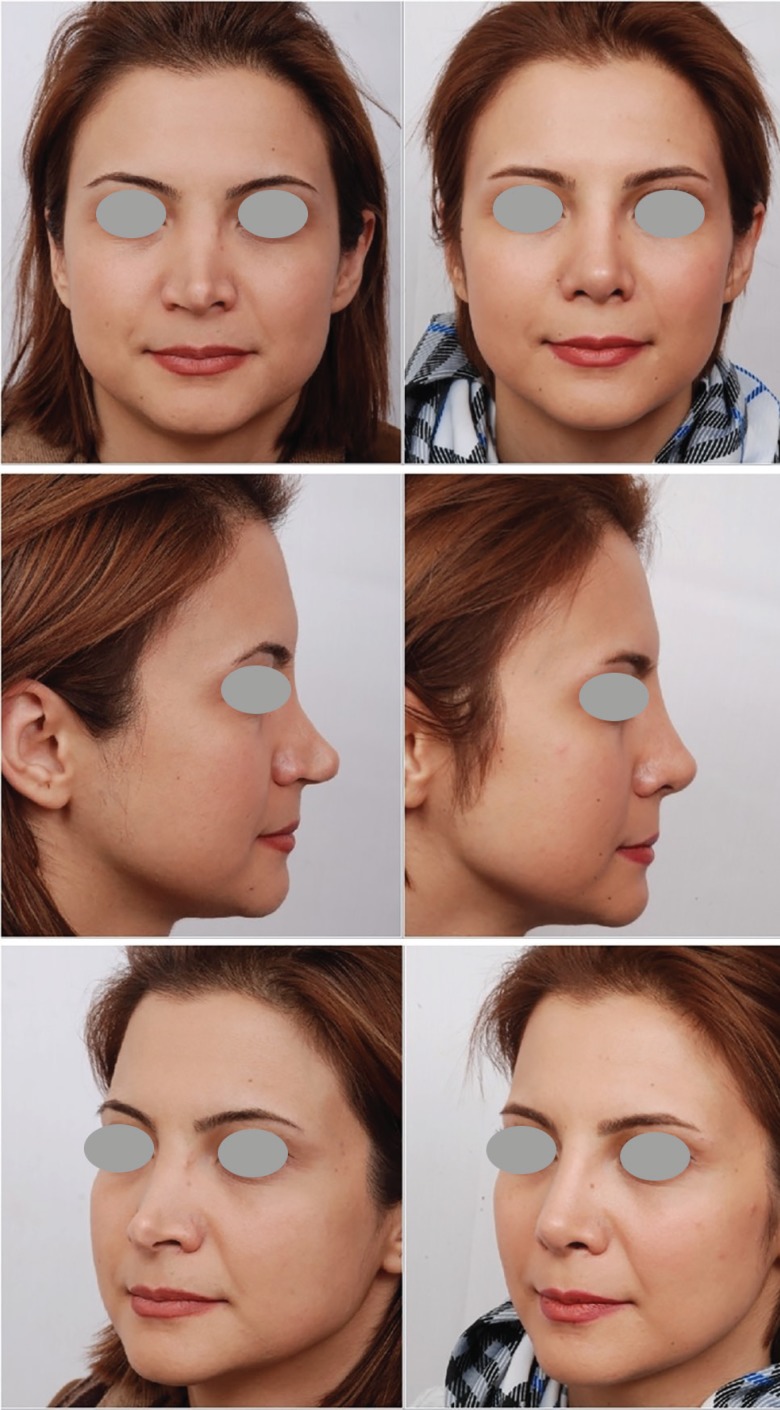
Secondary rhinoplasty with two previous unsuccessful surgeries, have been underwent skin massage and secondary rhinoplasty. Dorsal augmentation was performed with autologous grated cartilage from the source of rib cartilage wrapped in fascia. Results after 3 years

**Fig. 7:**
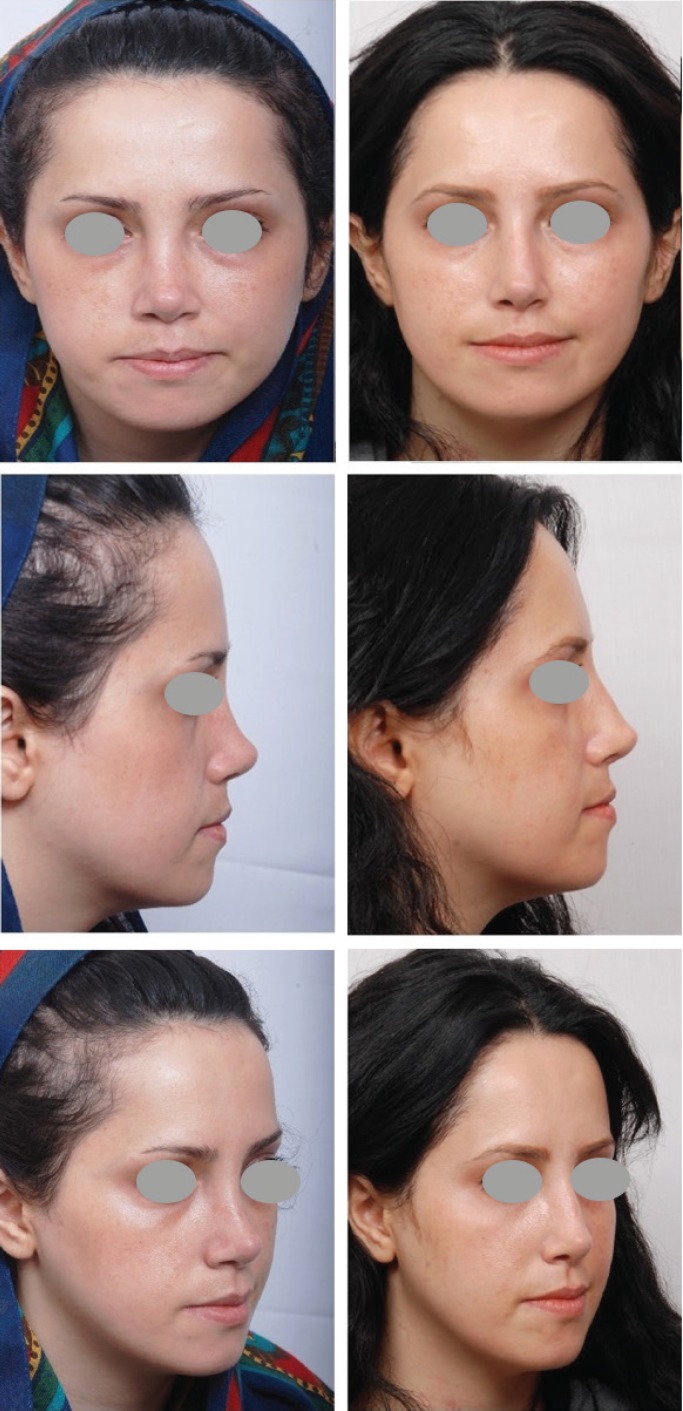
Secondary rhinoplasty with two previous surgeries. She complains from saddling of the nose. She had been undergone skin massage and secondary rhinoplasty with grated cartilage grafting of the dorsum. She has also some degree of overcorrection. The results are 2 years after surgery

**Fig. 8:**
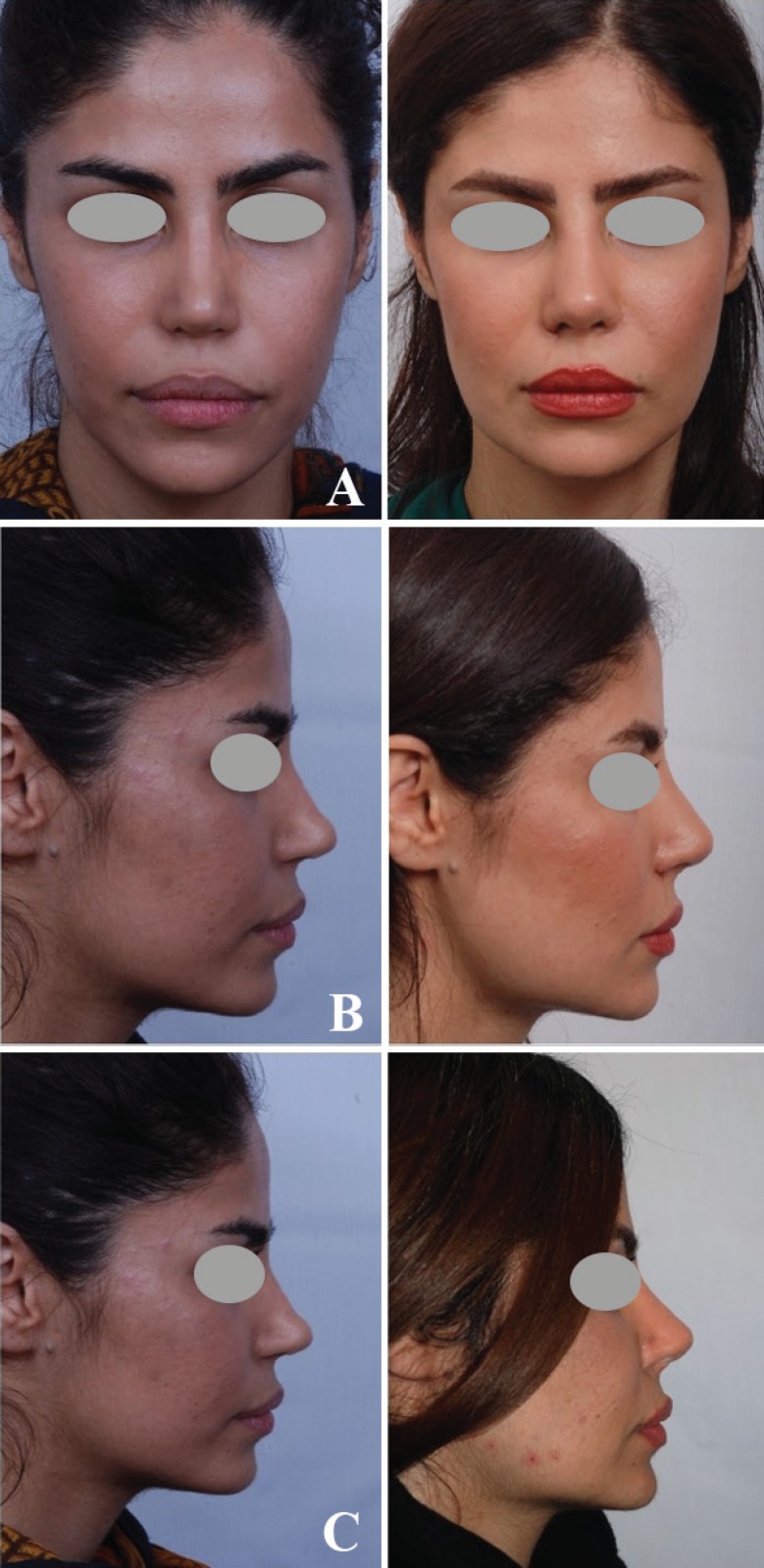
Case of 3 previous rhinoplasties had been undergone skin massage and corrective rhinoplasty with grated cartilage graft from the source of rib cartilage. Results (**A, B**) belong to 1 year after surgery and **C** is for 5 year post operative

**Fig. 9:**
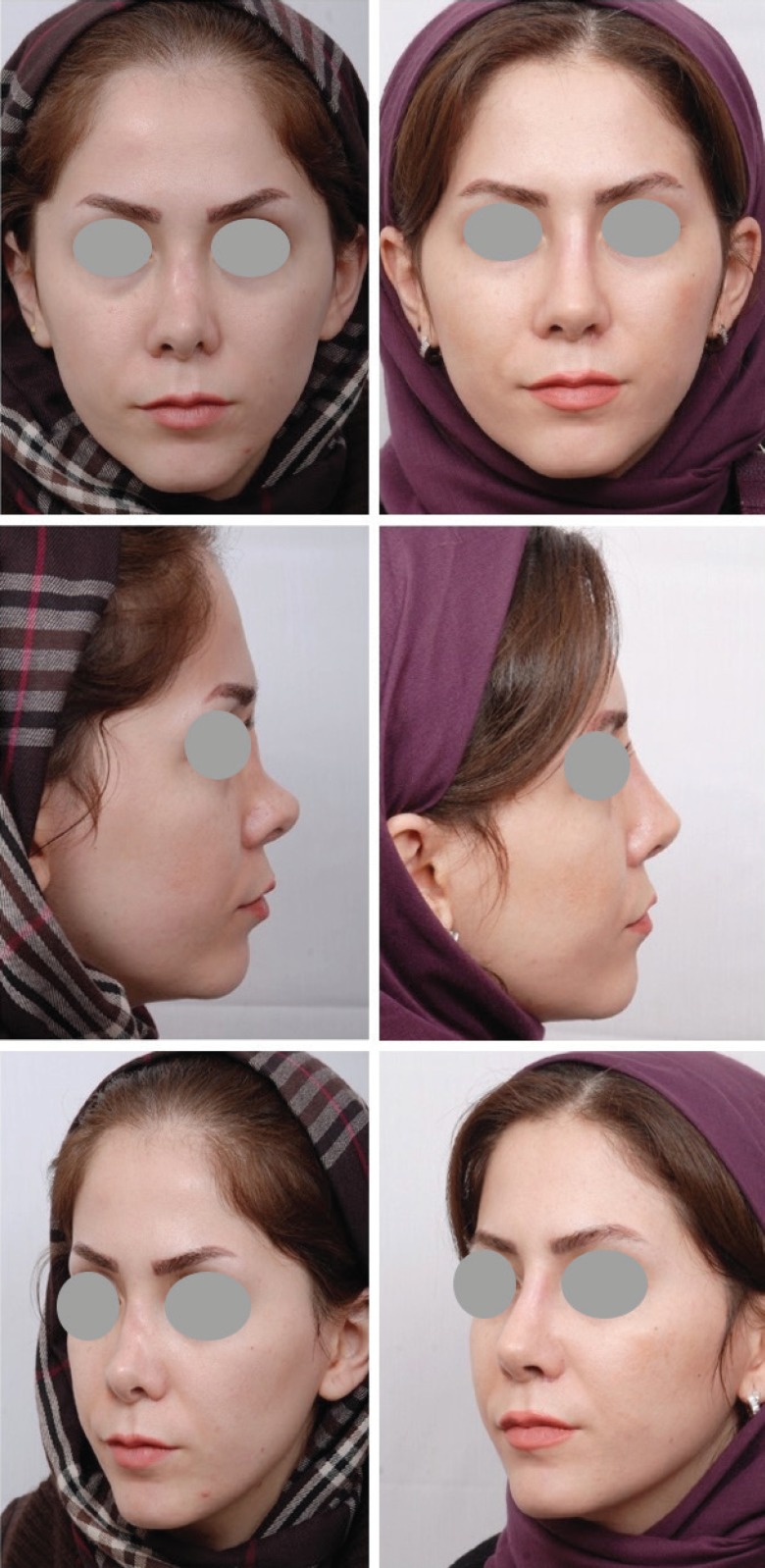
Secondary rhinoplasty case had 2 previous surgeries with marked saddling of dorsum which has been corrected with skin massage and grated cartilage graft. Results are 1.8 years post operative

**Fig. 10:**
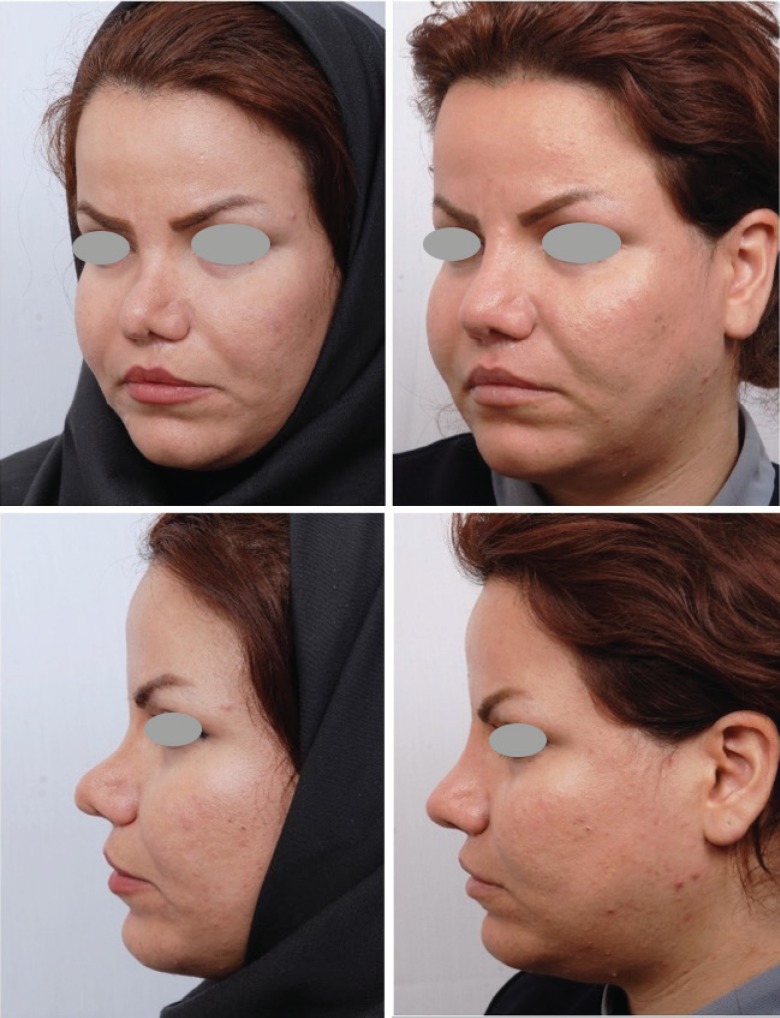
Case of secondary rhinoplasty with 2 previous surgeries on the nose and lip which was corrected with grated cartilage graft. The results are 2 years postoperative

**Fig. 11:**
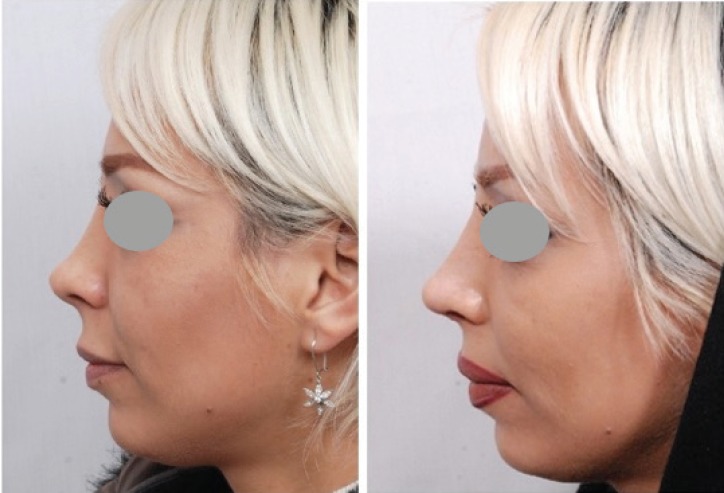
Case of secondary rhinoplasty with foreshortened nose which was corrected with skin massage and lengthening procedure and grated cartilage graft of the dorsum. The result belongs to 3 years postoperative

Overall, this technique resulted in acceptable satisfaction for the patients and surgeon. Except 23 cases (4.7%) who underwent revision, 460 patients (95.3%) needed no further intervention during a mean follow up of 2.8 years. Patients with a follow up period of less than 1 year, were excluded. Revision rate was 4.7% that is much better than usual revision rate in rhinoplasty. There are some pictures which shows some results and technical details (Figures 6-11)

## DISCUSSION

Any technique to mold and shape cartilage grafts in rhinoplasty, has advantages and disadvantages. Filler type cartilage grafts first known as crushed cartilage, were used to camouflage slight tip and dorsum irregularities since 1985^9^ and increasingly popularized till 1995^1^ and its use dramatically ceased recently.^10^ The use of diced cartilage grafts in reconstructive surgery was first described in 1943, though it was not for rhinoplasty. A number of studies describing diced cartilage have followed since then, but the technique had never achieved widespread use.^11^

In 2000, Erol introduced Turkish delight theory, using diced cartilage wrapped. Daniel and Calvert proved that Turkish delight is accompanied by a high resorption rate^5^ and introduced bare diced cartilage and DC-F grafts.^5^ For major soft tissue deficiencies and structural dorsal augmentation, DC-F graft is a well-known technique with a high degree of reliability.^1^ For slight soft tissue defects, diced cartilage can be used per se, but dicing process is technically difficult and operator dependent. It includes manually cutting a solid graft by surgical knife into pieces less than 1 mm in all dimensions. Cartilage segments are obviously not homogenous and it is a time-consuming process. Our grating device makes dicing process a fast and easy one with homogenous pieces. Shaved or grated pieces can be injected in an outpatient session to avoid a revision surgery.^8^ We have strong evidence about proper viability and retained volume of the grated cartilage with a very low revision rate (4.7%). 

We recommend our grated cartilage technique and grating device as a fast, easy and reliable method for cartilage molding and producing autogenous material to use as a natural filler for slight soft tissue defects in primary and secondary rhinoplasty. It can be accomplished both in an outpatient session and intraoperatively with a high degree of confidence. We persuade studies with longer periods of follow up and appreciate any expert commentary and criticism. According to the very low revision rate (less than 5%), we strongly recommend our grated cartilage graft for use in primary and secondary rhinoplasty. Our study showed that patient and surgeon`s satisfaction can be achieved with a high degree of confidence.

## Conflict of interest

The authors declare no conflict of interest.
